# Restoring the balance: immunotherapeutic combinations for autoimmune disease

**DOI:** 10.1242/dmm.015099

**Published:** 2014-05

**Authors:** Dawn E. Smilek, Mario R. Ehlers, Gerald T. Nepom

**Affiliations:** 1 The Immune Tolerance Network, 185 Berry Street #3515, San Francisco, CA 94107, USA.; 2 The Benaroya Institute, 1201 Ninth Avenue, Seattle, WA 98101, USA.

**Keywords:** Tolerance, Autoimmune, Biologic

## Abstract

Autoimmunity occurs when T cells, B cells or both are inappropriately activated, resulting in damage to one or more organ systems. Normally, high-affinity self-reactive T and B cells are eliminated in the thymus and bone marrow through a process known as central immune tolerance. However, low-affinity self-reactive T and B cells escape central tolerance and enter the blood and tissues, where they are kept in check by complex and non-redundant peripheral tolerance mechanisms. Dysfunction or imbalance of the immune system can lead to autoimmunity, and thus elucidation of normal tolerance mechanisms has led to identification of therapeutic targets for treating autoimmune disease. In the past 15 years, a number of disease-modifying monoclonal antibodies and genetically engineered biologic agents targeting the immune system have been approved, notably for the treatment of rheumatoid arthritis, inflammatory bowel disease and psoriasis. Although these agents represent a major advance, effective therapy for other autoimmune conditions, such as type 1 diabetes, remain elusive and will likely require intervention aimed at multiple components of the immune system. To this end, approaches that manipulate cells *ex vivo* and harness their complex behaviors are being tested in preclinical and clinical settings. In addition, approved biologic agents are being examined in combination with one another and with cell-based therapies. Substantial development and regulatory hurdles must be overcome in order to successfully combine immunotherapeutic biologic agents. Nevertheless, such combinations might ultimately be necessary to control autoimmune disease manifestations and restore the tolerant state.

## Introduction

Autoimmune diseases, such as rheumatoid arthritis (RA) and type 1 diabetes (T1D), have been defined as clinical syndromes that result from inappropriate activation of T cells, B cells or both, such that damage to one or more organ systems occurs (Davidson and Diamond, 2001). The immune system normally functions to recognize and defend against foreign pathogens by utilizing a highly diverse repertoire of specific immune receptors. A large number of these immune receptors recognize self-components, and must be eliminated or silenced by a process known as immune tolerance. The immune system, therefore, develops the ability to distinguish components of self from foreign invaders that need to be destroyed. A combination of genetic factors and environmental triggers can lead to disruption of immune tolerance. The underlying mechanisms are only partially understood, but could involve loss of balance between effector and regulatory components of the immune system (Bluestone, 2011). Restoration of the tolerant state is an important goal in the treatment of autoimmune diseases (Nepom et al., 2011). In this context, tolerance can be operationally defined as ongoing control of the manifestations of autoimmune disease following therapeutic intervention, without the need for chronic immunosuppressive medications.

Years of research in both animals and humans have led to the elucidation of many cellular and molecular mechanisms by which normal immune tolerance operates to prevent the onset of autoimmunity. A detailed discussion of tolerance mechanisms is beyond the scope of this article: these mechanisms have been reviewed in detail previously (Goodnow et al., 2005; Bluestone, 2011), and only a general overview of the elements of immune tolerance will be provided below. The results and conclusions of these studies have led to the identification of a large number of potential therapeutic targets, many of which are currently being explored in the preclinical or clinical trial setting. This Review will highlight selected examples, and will describe how combining these agents could more effectively restore the tolerant state in autoimmune disease.

## Mechanisms of immune tolerance and disruption in autoimmunity

**Fig. 1:**
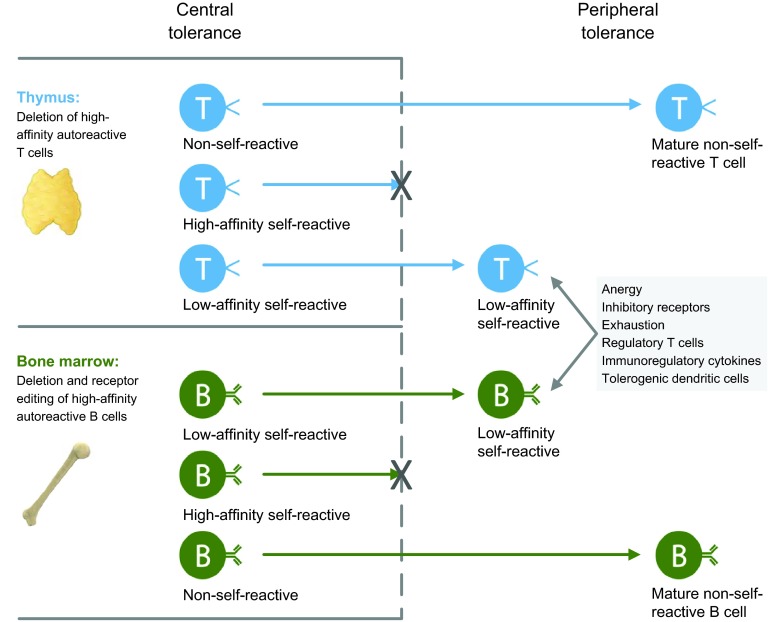
**Tolerance mechanisms in T cells and B cells.** Central tolerance occurs when high-affinity self-reactive T cells and B cells are eliminated in the thymus and bone marrow, respectively. Low-affinity self-reactive T cells and B cells escape central tolerance and enter the periphery, where they are kept in check by complementary and non-redundant peripheral tolerance mechanisms.

Our contemporary understanding of the normal development of immune tolerance can be dated to the 1940s, when hematopoietic chimerism was observed in fraternal cattle twins that shared a common circulatory system *in utero* (Owen, 1945). These cattle twins did not reject one another’s grafted skin, and subsequent work experimentally reproduced these findings in mice (Billingham et al., 1953). Extensive work in the intervening decades has shown that immune tolerance normally occurs by both central and peripheral mechanisms (Fig. 1). Central tolerance involves a complex developmental process whereby antigen-specific T and B cells (components of the adaptive immune system) are eliminated if they express high-affinity receptors for self-components. As detailed below, this occurs in the thymus (for T cells) and bone marrow (for B cells), and affects newly developing lymphocytes. Peripheral tolerance mechanisms come into play to suppress autoreactive T and B cells that have escaped into the periphery. Numerous mechanisms operate to maintain immune tolerance, involving multiple cell types and pathways that are designed to balance the need to avoid unwanted immune activation with the important need to maintain a diverse immune system. Indeed, pathogens frequently evolve virulence factors that take advantage of tolerogenic immune pathways specifically to evade immunity, providing selective pressures that could explain the evolution of so many complex and non-redundant tolerance mechanisms.

Within the immunological framework, there are several key points where functional balance is poised between a tolerant state and undesired immune reactivity, and that provide a guide to the variety of immune components that could be therapeutically targeted to restore a state of immune tolerance. T and B cells undergo tolerance by related but distinct mechanisms, which will be discussed separately. In addition, the important contribution to immune tolerance of innate immune cells, which lack antigen-specific receptors, will be discussed.

### Negative selection

**Fig. 2:**
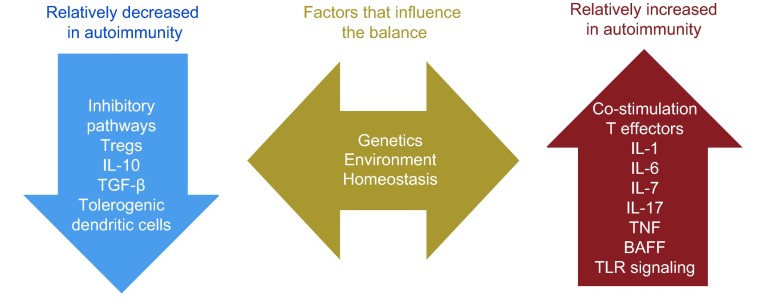
**Tolerance pathways are targets for immune intervention in autoimmune states.** A variety of mechanisms influence the balance of the regulatory and effector arms of the immune system. Strategies for treating autoimmunity target these pathways, such that insufficient regulatory mechanisms are enhanced and inappropriate activation mechanisms are diminished.

Negative selection of early developing autoreactive T cells occurs in the thymus and is dependent on the autoimmune regulator Aire, a transcription factor that promotes ectopic expression of tissue-specific antigens on medullary thymic epithelial cells (Anderson et al., 2002; Anderson and Su, 2011). This central tolerance mechanism allows T cells to encounter tissue-specific antigens in the thymus and undergo deletion. Defects in Aire are associated with the development of multi-organ autoimmune syndromes in both mice and humans (Nagamine et al., 1997; Aaltonen et al., 1997; Anderson et al., 2002; Ramsey et al., 2002; Anderson and Su, 2011). T cells that express low-affinity receptors for self-components escape negative selection, and join the mature T-cell repertoire. Thus, immune tolerance must be reinforced and maintained in the periphery by a number of additional mechanisms, including anergy, exhaustion and immune regulation (Goodnow et al., 2005; Bluestone, 2011). Each of these mechanisms has been studied in detail, leading to the identification of checkpoints that could be defective in autoimmunity (Fig. 2) and thus represent targets for immunotherapeutic intervention.

### T-cell anergy

T-cell anergy refers to the inactivation of lymphocytes, which undergo intrinsic molecular changes that prevent them from mediating effector functions (Jenkins and Schwartz, 1987; Choi and Schwartz, 2007). Specific recognition of antigen by T cells is generally understood to be necessary but not sufficient for T-cell activation, and additional co-stimulatory signals are required for T cells to develop effector status. A two-signal model for immune activation was proposed in 1970 (Bretscher and Cohn, 1970), and co-stimulatory molecular families required for activation of T cells were later identified and their functions characterized. Co-stimulatory signals are mediated by families of cell surface proteins such as CD28, and T-cell activation signals that occur in the absence of CD28 co-stimulation can result in T-cell anergy (Harding et al., 1992; Bour-Jordan et al., 2011).

### Inhibitory immunoregulatory signals

Cell surface molecules such as CTLA-4 and PD-1 deliver inhibitory immunoregulatory signals that are thought to be crucial to the maintenance of normal immune tolerance (Linsley et al., 1991; Krummel and Allison, 1995; Freeman et al., 2000; Carter et al., 2002; Watanabe and Nakajima, 2012; Okazaki et al., 2013). Cancer immunotherapy with antibodies against PD-1 and the corresponding PD-L1 ligand has recently been shown to be effective in treating certain types of cancer, presumably by potentiating the immune response to tumor antigens (Brahmer et al., 2012; Topalian et al., 2012; Wolchok et al., 2013). Successful interference in these inhibitory pathways in cancer trials is frequently accompanied by adverse autoimmune syndromes, however, such as inflammatory bowel disease or thyroiditis. This indicates a role for the CTLA-4 and PD-1 inhibitory molecules in normal tolerance mechanisms. Disruption of PD-1 in mice results in the development of a lupus-like autoimmune syndrome, and defects in PD-1 have been implicated in a number of other animal models of autoimmunity (Nishimura et al., 1999; Fife et al., 2006; Gianchecchi et al., 2013). The sustained expression of PD-1 is associated with T-cell exhaustion in the setting of chronic viral infection (Wherry et al., 2007; Virgin et al., 2009; Wherry, 2011), and exhaustion associated with PD-1 might support the maintenance of tolerance in low-affinity autoreactive T cells that escape deletion in the thymus and are chronically exposed to components of self in the periphery.

### Regulatory T cells

Regulatory T cells (Tregs) are another crucial component of peripheral tolerance mechanisms (Brusko et al., 2008; Sakaguchi et al., 2008; Rudensky, 2011). Many types of Tregs have been described, including ‘natural Tregs’, which develop in the thymus, and ‘induced Tregs’, which arise in the periphery. Natural Tregs express forkhead box protein 3 (Foxp3) and represent a distinct T-cell lineage that plays a major role in preventing autoimmunity. This is highlighted by the severe multi-organ autoimmune syndromes that develop when Foxp3 is deficient, such as the ‘scurfy’ phenotype in mice and the IPEX syndrome (immune dysregulation, polyendocrinopathy, enteropathy, X-linked) in humans (Bennett et al., 2001; Brunkow et al., 2001; Barzaghi et al., 2012). Foxp3-expressing Tregs usually represent a stable cell population that is crucial in maintaining immune tolerance but, under some conditions, these cells can undergo pathogenic conversion into T effector cells (Lal et al., 2009; Zhou et al., 2009; Bailey-Bucktrout et al., 2013; Komatsu et al., 2014). Although the clinical significance of Treg phenotypic instability in humans is uncertain (Bailey-Bucktrout and Bluestone, 2011; Sakaguchi et al., 2013), interleukin (IL)-17-producing Foxp3-expressing T cells have been observed in the synovium of individuals with RA, suggesting that these cells could be important contributors to the pathogenic autoimmune process (Joller and Kuchroo, 2014; Komatsu et al., 2014). Moreover, many immunosuppressive drugs that are used in clinical practice have the unintended effect of blocking Tregs as well as inhibiting effector components of the immune system, and there is a need to be more selective in order to fully exploit regulatory pathways in the maintenance of tolerance.

### Cytokines

Soluble factors or cytokines also play a role in the induction and maintenance of immune tolerance. IL-17 and interferon-γ have both inflammatory and homeostatic activities, and, when dysregulated, can promote autoimmunity. In contrast, IL-10 and TGF-β have predominantly regulatory effects in the context of inflammation and might be beneficial in maintaining tolerance and preventing autoimmunity (Surh and Sprent, 2012; Ng et al., 2013). IL-6 is a key cytokine that has been implicated in autoimmune disease; it supports the development of IL-17-producing T effector cells (Th17) and antagonizes the development of Tregs. This makes IL-6 a potentially powerful target for tolerance-generating therapies (Bettelli et al., 2006; Korn et al., 2008).

A number of other cytokines also have major roles in regulating immune function. T-effector and Treg survival and function are both dependent on IL-2, and the balance of the two opposing cell types could be influenced by the relative concentration of IL-2 or by an IL-2 signaling threshold effect that can lead to autoimmunity if disrupted (Tang et al., 2008; Boyman and Sprent, 2012). The homeostatic cytokine IL-7 supports T-cell survival under normal conditions (Schluns et al., 2000; Tan et al., 2001; Barthlott et al., 2003; Fry and Mackall, 2005). However, in the setting of lymphodepletion (loss of lymphocytes), IL-7 levels rise and could allow low-affinity autoreactive T cells to survive and proliferate when they otherwise would not (Kassiotis et al., 2003; Kieper et al., 2004; Goodnow et al., 2005; Tchao and Turka, 2012). Lymphodepletion and homeostatic expansion have been implicated in mouse and rat models of diabetes (Hornum et al., 2002; MacMurray et al., 2002; King et al., 2004), and could explain the association between autoimmunity and certain viral infections that can cause lymphopenia (abnormally low levels of lymphocytes).

### B cells: deletion or receptor editing

Developing B cells that express immunoglobulin receptors with high affinity for self-components also undergo negative selection, and are eliminated in the bone marrow through one of two central tolerance mechanisms: deletion or receptor editing (Nossal and Pike, 1975; Nemazee and Buerki, 1989; Gay et al., 1993). Receptor editing is a central tolerance mechanism by which the genes encoding the immunoglobulin receptors on developing high-affinity self-reactive B cells undergo recombination, such that the newly edited receptors lack high-affinity reactivity for self-components. However, B cells expressing immunoglobulin receptors with low affinity for self-components can escape deletion and receptor editing, and enter the peripheral circulation and lymphoid organs. An increased proportion of autoreactive B cells has been reported in T1D and other autoimmune conditions, as well as in healthy individuals who possess the *PTPN22* autoimmune susceptibility gene (Yurasov et al., 2005; Menard et al., 2011; Kinnunen et al., 2013a). Autoreactive B cells accumulate in the absence of functional Tregs in the IPEX autoimmunity syndrome (Kinnunen et al., 2013b), indicating that tolerance in the T- and B-cell compartments is interrelated.

### B-cell anergy

B cells with low-affinity receptors for self-components undergo B cell anergy, resulting in downregulation of surface IgM (Hartley et al., 1991). Anergic B cells display a high degree of autoreactivity, account for a large proportion of mature naïve B cells and seem to be abnormally activated in systemic lupus erythematosus (Quách et al., 2011). B-cell anergy is thought to result from chronic antigen-receptor occupancy (Gauld et al., 2005), which could occur in the presence of self-components that bind low-affinity B-cell antigen receptors. Defective regulation of B-cell receptor signaling has been implicated as a mechanism by which B-cell anergy fails in autoimmunity (Cambier, 2013; Dai et al., 2013).

### B-cell activating factor

The B-cell activating factor [BAFF; also known as B lymphocyte stimulator (BLyS)] family of cytokines contributes to B-cell tolerance by regulating B-cell development, selection and homeostasis (Cancro et al., 2009; Oropallo et al., 2011). Immature B cells can be more or less reliant for survival on BAFF, depending on the antigen-binding strength of their immunoglobulin receptor. By this mechanism, B cells expressing low-affinity receptors for self-components can be inappropriately preserved by the excess levels of BAFF present in autoimmune conditions such as systemic lupus erythematosus (Thien et al., 2004). BAFF could also influence homeostasis and tolerance in mature B cells, which can upregulate BAFF receptors in response to stimulation via Toll-like receptors (TLRs) (Groom et al., 2007; Treml et al., 2007). TLRs and B-cell immunoglobulin antigen receptors control activation of B cells through integrated signaling pathways, and TLRs have been linked to autoimmunity (Green and Marshak-Rothstein, 2011; Rawlings et al., 2012). It has been suggested that B-cell activation by the TLR pathway could allow pre-existing low-affinity autoreactive B cells to become effectors, and BAFF might be involved in this process (Oropallo et al., 2011).

### Innate immune system

Leukocytes that do not express antigen-specific receptors, such as dendritic cells, are components of the innate immune system. Immature dendritic cells recognize and become activated by pathogen- or damage-associated molecular patterns known as ‘danger’ signals via TLRs and other pattern-recognition receptors (Maldonado and von Andrian, 2010; Bao and Liu, 2013). These activated dendritic cells then stimulate the adaptive immune response by acting as co-stimulatory antigen-presenting cells for T cells (Bour-Jordan et al., 2011), and they also produce the inflammatory cytokines IL-1 and tumor necrosis factor (TNF). Immature dendritic cells, in contrast, can induce tolerance because they lack co-stimulatory cell surface markers and do not secrete the cytokines needed for T-effector-cell activation. Autoimmune syndromes develop in mice that lack immature dendritic cells (Ohnmacht et al., 2009; Bar-On and Jung, 2010). In addition, some subsets of dendritic cells, especially plasmacytoid dendritic cells, have been shown to induce Tregs (Liu, 2005; Maldonado and von Andrian, 2010).

Innate immune cells influence the development and maintenance of immune tolerance through direct or indirect interactions with T cells and B cells, and all three compartments can be involved in the autoimmune process. This point is illustrated by the protein tyrosine kinase Lyn-deficient mouse model, which develops a lupus-like autoimmune syndrome. In this model, T-cell-dependent autoimmune manifestations are controlled by altered dendritic cell signaling in combination with a defect in B-cell tolerance (Hua et al., 2014). Interaction between T cells, B cells and innate immune cells is likely to be the rule rather than the exception in autoimmune disease pathogenesis.

## Therapy for autoimmune disease

With so many elements of the immune system balanced between regulation and activation, successful restoration of tolerance in autoimmunity is likely to require intervention at a number of levels. These include dampening the innate immune response, deleting or disabling antigen-specific effector cells, and restoring or enhancing regulatory components of the immune system. As summarized below, the preponderance of prior therapeutic approaches have attempted to intervene by general immunosuppression or by targeting single molecules. Nonetheless, it is likely that more deliberate combinations of pathway-focused therapies will be needed to restore tolerance.

For many years, standard intervention in autoimmune disease consisted of diminishing autoimmune pathology by treatment with general immunosuppressive agents, anti-proliferative drugs (for example, mycophenolate mofetil) and corticosteroids. These traditional immunosuppressive agents continue to be first-line therapy for a number of autoimmune conditions such as lupus nephritis (Hahn et al., 2012); however, they are associated with high toxicity and incomplete efficacy. The introduction of disease-modifying monoclonal antibodies and genetically engineered biologic products, such as the TNF antagonist infliximab for RA and Crohn’s disease (Kornbluth, 1998; Lipsky et al., 2000), represented a major advance in the treatment of autoimmunity, because these agents generally act with greater specificity and lower toxicity than corticosteroids and other general immunosuppressive agents (St Clair, 2009).

Monoclonal antibodies and engineered fusion proteins are generally referred to as ‘biologic agents’, or simply ‘biologics’, and have become standard treatments in autoimmunity (Chan and Carter, 2010; Rosman et al., 2013; Yao et al., 2013), particularly when traditional disease-modifying drugs fail to control disease. For example, at least nine biologics are FDA-approved for the treatment of RA (Malviya et al., 2013), and several biologics are also approved for the treatment of psoriasis and inflammatory bowel disease, among others (Table 1). In addition, the approved biologic agents are sometimes utilized ‘off label’ for non-approved indications when autoimmune disease proves refractory to standard therapy (Hahn et al., 2012), or in autoimmune diseases for which no effective approved therapy exists (Greenberg et al., 2012). Biologic agents intervene in autoimmunity by a variety of mechanisms, including cytokine blockade, depletion of T or B cells and immunomodulation. In addition to the agents listed in Table 1, many new biologic agents are currently in development (Chan and Behrens, 2013; Reichert, 2013; Yao et al., 2013). In addition, a number of novel small molecules have been discovered that target the immune system. One notable example is tofacitinib, an inhibitor of the Janus kinase (JAK)-receptor-associated signaling pathway, which is involved in cytokine-mediated activation of the immune system and is potentially a central contributor to autoimmune pathogenesis (Furumoto and Gadina, 2013).

**Table 1:**
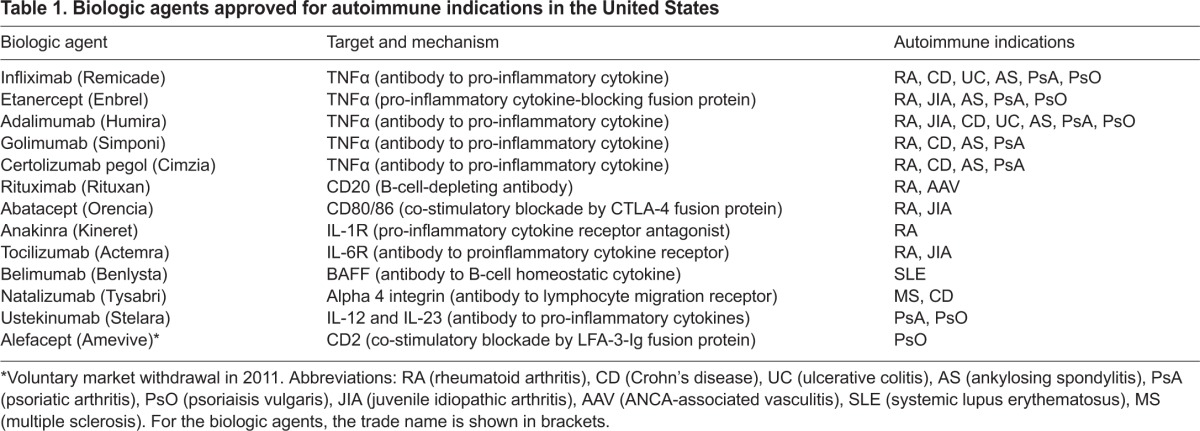
Biologic agents approved for autoimmune indications in the United States

Even relatively specific biologic agents can be associated with negative consequences, such as vulnerability to infection, and it is a challenge to intervene in autoimmune disease without overly compromising the normal immune response. Moreover, none of the biologics have been shown to restore immune tolerance to the extent that disease remains quiescent after therapy is withdrawn. The immune system is a complex network of cellular pathways and cytokine interactions, and the therapeutic approach with biologics has been to inhibit one immune pathway based on a rational understanding of disease pathogenesis. But, in many cases, this has proven insufficient to fully control autoimmunity and thus additional strategies are needed.

As an example, T1D is a disease in which the underlying autoimmune pathogenesis has been extensively explored in both mice and humans. This understanding has led to interventional trials with biologic agents aimed at preserving insulin-secreting β-cells early in the course of the disease (Bluestone et al., 2010). Treatment of T1D with teplizumab or otelixizumab – monoclonal antibodies to CD3, which is present on T cells – transiently preserved insulin-secreting function in some subjects (Herold et al., 2005; Keymeulen et al., 2010; Herold et al., 2013b). Similarly, preservation of insulin secretion in T1D has been observed following B-cell depletion with an anti-CD20 monoclonal antibody, rituximab (Pescovitz et al., 2009), and also following co-stimulatory blockade with a CTLA-4 fusion protein, abatacept (Orban et al., 2011). However, in each of these studies, most participants experienced a recurrence of progressive β-cell loss, indicating that tolerance was not achieved. Thus, insulin replacement therapy remains the central feature of T1D management, as it has been for decades.

New therapeutic strategies for treating T1D are being considered, including biologic agents that target the cytokines IL-1, TNF, IL-12p40, IL-17 and IL-6 (Herold et al., 2013a; Nepom et al., 2013). Preclinical experiments in animal models and early clinical studies support further evaluation of these agents in clinical trials. For example, experiments in mice indicate that IL-6 induces methylation of Foxp3 in Tregs, resulting in a loss of regulatory function (Lal et al., 2009). Pathogenic ‘exTreg’ populations have been identified in association with autoimmune responses in mice (Zhou et al., 2009; Bailey-Bucktrout et al., 2013), and recent studies in a murine arthritis model provide further evidence that Tregs can undergo pathogenic conversion to Th17 cells (Komatsu et al., 2014). Together, the results suggest that a monoclonal antibody that targets the IL-6 receptor might prevent pathogenic conversion of Tregs and re-establish regulatory function in T1D, thereby interrupting immune-mediated destruction of insulin-secreting β-cells.

Using another approach, promising results were recently obtained in a T1D trial with alefacept, a fusion protein that targets CD2 expressed on effector memory T cells and central memory T cells (Rigby et al., 2013a). Key clinical end points were met in this study, and accompanying mechanistic studies demonstrated depletion of effector and memory T cells, without elimination of Tregs. This study supports the concept that restoring the proper balance of regulatory- and effector-cell compartments can be an effective strategy for treating autoimmune disease. One potential approach to achieving this goal could be a combination of two agents: one that enhances Tregs by interfering with the IL-6 pathway, and the other agent targeting effector and memory T cells.

## Cell-based therapy for autoimmune disease

Although biologics are a solidly established component of the pharmaceutical armamentarium, novel cell-based therapeutic approaches are also being developed for the treatment of autoimmune disease. Cells can be highly specific, self-perpetuating and subject to intrinsic regulatory mechanisms, unlike traditional immunosuppressives and the newer biologic agents. The pharmaceutical development pathway for cell-based therapeutics is not straightforward because it will require individualized expansion and manipulation of autologous cells under good manufacturing practice (GMP) conditions. Moreover, the function of these cell populations will need to be predictable once administered. Extensive translational research is still required. Nevertheless, cells are capable of complex sets of behaviors and, in some cases, have proven amenable to the type of cellular engineering required to achieve the desired therapeutic effect (Fischbach et al., 2013; Kalos and June, 2013).

As discussed above, Tregs and other regulatory immune cell compartments are central to immune tolerance (Brusko et al., 2008; Murphy et al., 2011; Rudensky, 2011; Kalampokis et al., 2013). The ONE Study, a large-scale international collaboration, is evaluating the use of Tregs, dendritic cells, macrophages and other regulatory cell types for their potential use in solid organ transplantation tolerance (Geissler, 2012; Riquelme et al., 2012). Manipulation of T cells, B cells or the innate immune compartment is also a potential approach for treating autoimmune disease, and has been demonstrated in several animal models of autoimmunity. *Ex vivo* expansion and transfer of Tregs into diabetes-susceptible NOD mice prevented and reversed diabetes (Bluestone and Tang, 2004; Tang et al., 2004; Jaeckel et al., 2005), and suppressed renal disease in lupus-prone NZB/NZW mice (Scalapino et al., 2006). In a murine model of myelin-peptide-induced experimental autoimmune encephalomyelitis (EAE), adoptive transfer of Tregs conferred protection against the development of central nervous system inflammation and clinical signs of disease (Kohm et al., 2002). Treg transfer also reversed established lamina propria infiltrates and restored normal intestinal architecture in an experimental murine model of inflammatory bowel disease (Mottet et al., 2003).

In humans, purified Tregs from patients with recent-onset T1D and from healthy individuals have been successfully expanded *ex vivo* using IL-2 and microbeads coated with anti-CD3 and anti-CD28 (Putnam et al., 2009). The resulting population of Tregs displayed functional properties and showed stable expression of regulatory-cell markers and cytokines. These results represent an important first step in developing a personalized therapeutic Treg product for T1D, and potentially other autoimmune diseases (Esensten et al., 2009; Thompson et al., 2012; Herold et al., 2013a). Nonetheless, further information is needed regarding the persistence of *ex vivo* expanded Tregs and their capacity to maintain their regulatory phenotype following transfer (Putnam et al., 2009; Bailey-Bucktrout and Bluestone, 2011; Joller and Kuchroo, 2014).

## Antigen-specific immunotherapy for autoimmune disease

Antigen-specific tolerance can be induced when antigen is introduced under tolerogenic rather than immunogenic conditions. In practical terms, antigen introduced by the oral route or in soluble form tends to diminish rather than potentiate subsequent immune responses to that antigen. Antigen-specific tolerance is the basis for immunotherapy with allergen extracts to treat allergic disease, and has been advanced as a potential approach to treating autoimmune disease. An antigen-specific immunotherapeutic approach would allow protective immune cells to remain intact and maintain their normal immune surveillance functions, while specifically targeting the cells thought to be responsible for disease pathogenesis. This concept has been explored in T1D, in which the candidate autoantigens glutamic acid decarboxylase and insulin are effective in treating diabetes in NOD mice (Zhang et al., 1991; Tian et al., 1996; Tisch et al., 1999). Administration of these candidate autoantigens has so far failed to prevent or reverse T1D in humans (Peakman and von Herrath, 2010; Coppieters et al., 2013), although modest improvement in insulin C-peptide levels was observed following administration of a proinsulin-encoding plasmid in T1D (Roep et al., 2013).

Identification and disarming of pathogenic disease-specific cells is challenging when the precise antigenic target of the pathogenic cells is not known. For this reason, murine models of induced disease have been crucial in understanding and modeling intervention in the pathogenic process. Antigen-specific tolerance has been extensively explored in murine EAE, in which mice develop a relapsing demyelinating syndrome following immunization with purified myelin components. In the EAE model, administration of soluble myelin peptide prevents and reverses disease in mice when the therapeutic peptides correspond to myelin protein sequences that are most responsible for disease (Wraith et al., 1989; Smilek et al., 1991; Gaur et al., 1992; Metzler and Wraith, 1993; Samson and Smilek, 1995). Although some encouraging results have been reported in humans (Warren et al., 2006), overall this strategy has not been successful in clinical trials (Bielekova et al., 2000; Kappos et al., 2000; Garren et al., 2008; Freedman et al., 2011), and has been associated with hypersensitivity and anaphylactic reactions in some circumstances (Kappos et al., 2000; Smith et al., 2005). There are a number of possible explanations for why promising results with antigen-specific therapy in mice have not led to demonstration of efficacy in clinical trials (Box 1).

**Box 1. The mouse trap – using mice as a preclinical testing ground for immunotherapies**

Animal models, particularly mouse models, have been crucial to our understanding of immunology and immune tolerance, as evidenced by the many mouse model studies that are highlighted in this Review. Development of all of the biologics listed in Table 1 has depended on invaluable basic research conducted in mice. Mice are small, inexpensive, easy to maintain, and reproduce rapidly. The commercial availability of many inbred mouse strains has ensured a ready supply of individual mice that are genetically identical to one another, yet distinct from other mouse strains. The power of such a resource in the study of immune tolerance cannot be underestimated. Moreover, mice have proven amenable to genetic manipulation, allowing immunologists to create their own lines of transgenic mice and knockout mice deficient in specific genes, using procedures that have become standard.

Nevertheless, there is a limit to what can be accomplished in mice in terms of predicting a therapeutic result in human autoimmune disease. Promising results with antigen-specific immunotherapy in diabetic NOD mice and myelin-induced murine experimental autoimmune encephalomyelitis (EAE) so far have not been translated into effective therapies for T1D or multiple sclerosis in humans. A number of explanations for these disappointing results have been discussed previously, and serve as a guide to what we can reasonably expect to learn from mice (von Herrath and Nepom, 2005; Davis, 2008; Couzin-Frankel, 2013). Mouse strains are highly inbred, each an essentially identical genetic copy of the next, and therefore their greatest strength for the study of basic immunology is also their greatest drawback as a model for clinical trials, which must be conducted in genetically diverse human beings. Exploratory studies in small numbers of mice are conducted in single laboratories rather than multiple centers, with few exceptions (Sarikonda et al., 2013). These experiments generally are unblinded, and utilize animals which have not undergone proper randomization. Moreover, translation of promising results in mice might fail owing to the challenges inherent in optimizing dose, frequency, route of administration and vehicle or adjuvant in the clinic. In summary, mice contribute to our understanding of autoimmunity and the development of potential interventions for autoimmune disease, but cannot be solely relied upon to predict the outcome of clinical trials in humans.

An alternative cell-based approach for treating autoimmune disease involves antigen-specific tolerance induction with chemically fixed cells to which self-peptides have been coupled. Administration of myelin peptides that have been chemically coupled to leukocytes with ethylene carbodiimide (EDCI) is both safe and effective in treating EAE in mice (Turley and Miller, 2007; Getts et al., 2011). The mechanism by which peptide-coupled EDCI-fixed cells induces tolerance involves uptake of the apoptotic fixed cells by macrophages, IL-10 production, regulation of the negative co-stimulatory molecule PD-L1, and induction of Tregs (Turley and Miller, 2007; Getts et al., 2011). An early-stage trial in multiple sclerosis has shown this strategy to be safe in humans, and reduction in T-cell responses to myelin peptides has been demonstrated using this approach, an important first proof-of-concept step in demonstrating that a therapeutic result can be achieved using myelin-peptide-coupled cells (Lutterotti et al., 2013).

Although promising, the practicality of individualized production of antigen-coupled isologous leukocytes under GMP conditions limits broad clinical application of cell-based antigen-specific tolerance. This problem would be reduced by coupling tolerogenic peptides to inert nanoparticles (Tyner and Sadrieh, 2011; Getts et al., 2012). In the EAE model, peptide-coupled microparticles were effective in preventing and treating disease by targeting a natural apoptotic clearance pathway that requires the scavenger receptor MARCO, and which leads to activation of Tregs, abortive T-cell activation and T-cell anergy (Getts et al., 2012). These cell-based and nanoparticle approaches are potentially applicable to other autoimmune conditions in which pathogenic self-antigens have been identified.

## Combination therapy for autoimmune disease

As discussed above, multiple disease-modifying monoclonal antibodies and biologic agents have been developed and approved for the treatment of autoimmune disease, with many more currently in development. Furthermore, cell- and nanoparticle-based therapies for autoimmune disease might be available in the future. Because a variety of non-redundant mechanisms contribute to the maintenance of normal immune tolerance, the next logical step is to combine therapies in order to potentiate efficacy in autoimmune disease. Thorough understanding of the mechanism of action of each agent is crucial to choosing rational combinations, and it is equally important to formulate hypotheses about why a particular biologic agent fails to adequately treat disease when used alone. Two or more complementary and non-redundant pathways could be targeted in sequence, to first induce autoimmune disease remission, and then restore a state of immune tolerance and prevent reactivation of the disease process.

Once the autoimmune process has been triggered, a detrimental cascade of events occurs, leading to the downstream activation of pathogenic cell populations and the production of pro-inflammatory cytokines. Monoclonal antibodies and recombinant fusion proteins that target pro-inflammatory cytokines such as TNF, IL-1, IL-6, IL-12 and IL-23 are extremely effective for some autoimmune indications (Table 1), and agents that target IL-17 are in development. However, the immune cells that produce these pro-inflammatory cytokines, as well as cells that are responsive to them, are not eliminated by the anti-cytokine treatment, so can be reactivated when the biologic agents are removed, resulting in recurrent disease (Leonardi et al., 2008). In other cases, monoclonal antibodies that eliminate presumed pathogenic immune cells exert their effect for a limited period of time. Eventual reconstitution of the pathogenic cell compartment also results in recurrent disease (Lazarus et al., 2012).

**Fig. 3:**
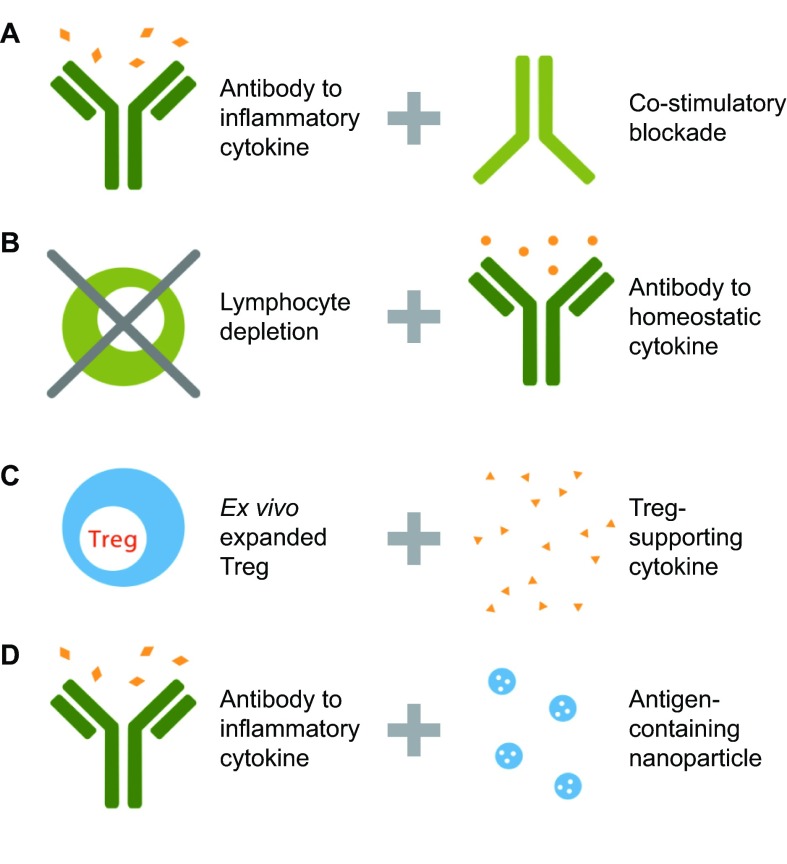
**Examples of combination therapy for autoimmunity.** Successful treatment of autoimmune disease is likely to require novel combinations of pathway-targeted cell-based therapies and biologic agents. (A) Manifestations of autoimmune disease could be reduced with an anti-inflammatory cytokine, and recurrence prevented by co-stimulatory blockade. (B) Pathogenic T or B cells could be depleted, and re-emergence of autoreactive cells during homeostatic proliferation could be prevented by an antibody that targets a homeostatic cytokine. (C) Tregs that have been expanded *ex vivo* could be infused with a cytokine that prevents their pathogenic conversion to an inflammatory phenotype. (D) Manifestations of autoimmune disease could be reduced with an anti-inflammatory cytokine, in combination with antigen-specific tolerance induction using antigen-containing nanoparticles.

One strategy for solving the problem of recurrent autoimmune manifestations is to add a second biologic agent that induces tolerance in effector cells undergoing reactivation or reconstitution. Examples of this strategy are illustrated in Fig. 3. Choice of the second biologic agent should be based on an understanding of normal mechanisms of immune tolerance. T-cell tolerance via induction of anergy can be induced in murine models by blockade of the co-stimulatory CD28 pathway, so a co-stimulation blocking agent would be a logical second biologic (Fig. 3A) in diseases in which a T-cell component is thought to be important, such as psoriasis (Mueller et al., 1989; Kremer et al., 2005; Bour-Jordan et al., 2011). The B-cell tolerance threshold is influenced by BAFF, such that higher levels of BAFF allow the persistence of autoreactive B cells. Therefore, an agent that blocks BAFF could be a second biologic (Fig. 3B) in diseases in which B cells are thought to contribute, such as systemic lupus erythematosus (Thien et al., 2004; Cancro et al., 2009; Navarra et al., 2011). In both these examples, autoimmune disease remission would be induced by first depleting or antagonizing the relevant pathogenic cell population or pro-inflammatory cytokine pathway. Once the inflammatory immune process has been interrupted, the second biologic would then be introduced to prevent reactivation of the autoimmune disease process such that the pathogenic cascade does not recur.

Another possibility would be to combine a cell-based Treg therapy with a biologic agent that maintains the desired functional state of Tregs (Tang et al., 2008), or which antagonizes the development of T-effector cells, perhaps by interfering with the IL-6 pathway (Fig. 3C). Finally, monoclonal antibodies and other biologic agents could be combined with an antigen-specific approach, a concept that has been proposed for T1D because of the potential to reduce toxicity associated with the former (Matthews et al., 2010). For example, an anti-inflammatory biologic agent could be added to antigen-containing nanoparticles, potentiating their tolerogenic effects (Fig. 3D) (Fraser, 2010; Getts et al., 2012). Many other rational combinations are possible beyond those illustrated in Fig. 3.

It should be noted that more than two agents might be needed to fully control autoimmune disease, because T cells, B cells and innate immune cells all contribute to the autoimmune process, as described above. Diseases such as T1D and lupus nephritis, which have proven refractory to treatment with standard immunosuppression and single biologic agents, might require a more complex approach using combinations that target multiple immune pathways. Combinations could be selected that: (1) eliminate effector T and B cells, (2) target antigen-specific cells, (3) expand Tregs, and (4) dampen the innate immune response and inflammation. Moreover, agents that are effective early in the course of disease might not be as useful later, when the disease process is fully established. Immune intervention prior to the onset of overt autoimmune disease is being explored in T1D, for example, but depends on the ability to predict the risk of developing this disease with a high degree of certainty.

Although promising for the successful treatment of autoimmune disease, combining biologic agents and cell-based or antigen-specific therapies involves addressing a number of special challenges, some of which have been discussed previously (Matthews et al., 2010; Nepom et al., 2013). With these challenges in mind, an outline of feasible combination immunotherapies for T1D has been developed and published (Matthews et al., 2010). Identifying the correct combination of agents is not straightforward. In some cases, the particular combination might be required to achieve the intended biologic effect, even if the individual agents have no demonstrated efficacy signal when used alone. Ideally, however, therapies with proven individual safety and efficacy profiles would be chosen for a combination. Even so, the clinical development and regulatory pathway for combinations will be complex if separate pharmaceutical companies are required to partner in order to obtain an approved indication for the combination.

Of primary concern is the safety of combining two biologics that target complementary and non-redundant immune pathways. Even if two agents with well-established safety profiles are combined, the potential still exists for additive or synergistic deleterious effects. In a recent study, the safety profile of the B-cell-depleting monoclonal antibody rituximab added to other biologics in RA was similar to the standard prescribed combination of rituximab and methotrexate (Rigby et al., 2013b), indicating that some combinations of biologics have an acceptable safety profile. However, an increase in serious infections was observed when the IL-1-receptor antagonist anakinra was added to the anti-TNF agent etanercept in RA (Genovese et al., 2004), and also when other biologics were added to the CTLA-4 co-stimulatory blocking agent abatacept (Weinblatt et al., 2006). In contrast, the ARRIVE trial showed that participants with RA could be safely switched from TNF antagonists to abatacept without a washout period, i.e. an interceding period of no treatment (Schiff et al., 2009; Genovese et al., 2012). Similarly, in psoriasis, individuals can be safely switched without a washout period from the TNF agent etanercept to ustekinumab, a monoclonal antibody that targets IL-12 and IL-23 (Griffiths et al., 2010). These later study results support the safety of sequential combinations of two biologics.

Another issue is the variability in responsiveness of individual patients to biologic agents. Baseline levels of interferon gene expression are associated with responsiveness to β-interferon in multiple sclerosis (Verweij and Vosslamber, 2013), and an interferon signature has also been associated with responsiveness to rituximab in RA (Raterman et al., 2012). In T1D, baseline hemoglobin A1C and insulin usage predicted relative insulin C-peptide preservation in response to the anti-CD3 monoclonal antibody teplizumab in a subpopulation of treated participants (Herold et al., 2013b). This variability in individual responsiveness can be foreseen when biomarkers allow personalized targeting of therapeutic agents to appropriate individuals, such as the use of periostin as a marker for asthma in individuals who are likely to respond to anti-IL-13 (lebrikizumab) (Corren et al., 2011; Chan and Behrens, 2013). When biologics are combined, the complexity of response patterns is likely to be increased, and biomarkers that predict responsiveness are lacking in most circumstances. Sufficient numbers of participants will need to be enrolled to adequately power these complex clinical trials, and special attention to mechanistic studies will be required so that relevant biomarkers can be identified.

## Conclusions

Significant advances have been made in the treatment of autoimmunity using disease-modifying drugs and biologics in conditions such as RA, inflammatory bowel disease, psoriasis and multiple sclerosis. However, therapeutic responses associated with these approaches are not durable, and require long-term continuation of therapy. Moreover, most subjects with T1D who have been treated with immunomodulation have shown a transient rather than sustained response. Potent general immunosuppression, which is associated with toxicity and is not fully effective, remains the standard of care for other conditions, such as lupus nephritis. Substantial unmet medical need exists for these conditions. Successful restoration of immune tolerance will require innovative approaches utilizing rational targeting of multiple immunological pathways.
